# Detection, Composition and Treatment of Volatile Organic Compounds from Waste Treatment Plants

**DOI:** 10.3390/s110404043

**Published:** 2011-04-06

**Authors:** Xavier Font, Adriana Artola, Antoni Sánchez

**Affiliations:** Composting Research Group, Department of Chemical Engineering, Universitat Autònoma de Barcelona, 08193-Bellaterra (Cerdanyola del Vallès), Barcelona, Spain; E-Mails: xavier.font@uab.cat (X.F.); antoni.sanchez@uab.cat (A.S.)

**Keywords:** volatile organic compounds (VOC), organic waste, waste treatment plants, composting, anaerobic digestion, gaseous emissions, odor

## Abstract

Environmental policies at the European and global level support the diversion of wastes from landfills for their treatment in different facilities. Organic waste is mainly treated or valorized through composting, anaerobic digestion or a combination of both treatments. Thus, there are an increasing number of waste treatment plants using this type of biological treatment. During waste handling and biological decomposition steps a number of gaseous compounds are generated or removed from the organic matrix and emitted. Different families of Volatile Organic Compounds (VOC) can be found in these emissions. Many of these compounds are also sources of odor nuisance. In fact, odors are the main source of complaints and social impacts of any waste treatment plant. This work presents a summary of the main types of VOC emitted in organic waste treatment facilities and the methods used to detect and quantify these compounds, together with the treatment methods applied to gaseous emissions commonly used in composting and anaerobic digestion facilities.

## 1. Organic Waste Treatment Plants and Gaseous Emissions

Solid waste management is becoming a global problem in developed countries. According to international recommendations and legislation, different technologies are being used to reduce landfill disposal of organic wastes, improving recycling of organic matter and nutrients [1]. Among the technologies used to treat the source-separated organic fraction of municipal solid wastes (OFMSW), anaerobic digestion and composting are widely recommended as environmentally friendly technologies. Regarding this, although the objective of composting and anaerobic digestion, as well as other waste treatment technologies, is to safely transform wastes into less polluting and/or hazardous substances or, when possible, useful products reducing their possible impact on the environment, there are some inherent environmental impacts associated with waste recycling in large-scale facilities. Odorous compound emissions and atmospheric pollution are the most common of these impacts. In fact, composting plants present numerous odor and pollution sources, including reception and materials handling, forced aerated composting, stockpiling, *etc*. Gaseous emissions in composting facilities are typically constituted by nitrogen-based compounds, sulphur-based compounds and a wide group of compounds denominated Volatile Organic Compounds (VOCs) [2]. VOCs are also produced during the anaerobic digestion process [3], although their composition can be very different from that of aerobic processes.

Volatile Organic Compounds is a denomination used to refer to a wide group of organic compounds whose vapor pressure is at least 0.01 kPa at 20 °C [4]. VOCs are also characterized by their low water solubility. Once in the atmosphere, VOCs participate in photochemical reactions producing photochemical oxidants. According to Eitzer [2], who undertook a pioneering study on the exhaustive characterization of the different VOCs emitted at the different stages of the composting process, most VOCs in composting plants are emitted during the early stages of process: *i.e*., at the tipping floors, at the shredder and during the initial forced aeration composting period. Also, incomplete or insufficient aeration during composting can produce sulphur compounds of intense odor, whereas incomplete aerobic degradation processes result in the emission of alcohols, ketones, esters and organic acids [5]. Van Durme *et al*. [6] identified dimethyl sulphide, dimethyl disulphide, limonene and α-pinene as the most significant odorous VOC in a wastewater sludge composting facility. According to this study, the latter two compounds were released from the wood chips used as bulking agent. In fact, Büyüksönmez and Evans [7] also found six different terpenes (α-pinene, β-pinene, 3-carene, camphene, β-myrcene and D-limonene) as the major compounds responsible for VOC emissions from green waste composting processes (within 33 and 95% of total emissions). These compounds were also predominant in feedstock natural breakdown.

Delgado-Rodríguez *et al*. [8] reported that volatile compounds emissions are closely related to the composting process phases: aldehydes, alcohols, carboxylic acids, esters, ketones, sulphides and terpenes are mainly emitted during the initial acid phase, while in the thermophilic phase ketones, organosulphur-compounds, terpenes and ammonia become predominant. During the cooling phase sulphides, terpenes and ammonia are the main volatile compounds emitted. These authors also investigated the effect of the process control parameters (moisture, aeration and C/N ratio) on volatile compound emissions in MSW (Municipal Solid Waste) composting. The C/N ratio had the most significant effect on VOC emissions, followed by the aeration level and the moisture content. The effect of aeration on emissions from composting mixtures was also highlighted by Staley *et al*. [9], when comparing emissions from aerobic and anaerobic treatments. However, in both cases it must be considered that the C/N ratio should be related to the biologically available carbon and nitrogen forms, a point that is not often considered [10]. At the laboratory scale, total VOC concentration in exhaust gases from composting processes of different wastes has been also studied and it was concluded that the highest concentrations of VOCs were emitted during the first 48 h of the process [11]. These authors also stated that total VOC emissions could not be correlated with the biological activity of the process as measured by respirometry. Pierucci *et al*. [12] found the same emission pattern (main VOC release during the first days of process), although the possible correlation with the biological activity was not studied.

Tolvanen *et al*. [13] and Komilis *et al*. [14] studied VOC emissions from the composting process considering the possible negative effects on plant workers and the nearby population. Tolvanen *et al*. [13] stated that VOC concentrations detected at a composting plant treating source-separated catering waste were much lower than the national threshold limit values, thus avoiding primary health effects, although many of the compounds exceeded their threshold odor concentrations; thus secondary symptoms as nausea and hypersensibility might be expected from exposure to unpleasant odors. In fact, the effect of workers’ simultaneous exposure to several VOCs is not very well known since synergic effects should be also considered. On the other hand, Komilis *et al*. [14] found various xenobiotic VOCs in gaseous emissions during laboratory scale composting of different MSW components.

VOC emissions in biological treatment processes have also been studied as environmental loads related to these processes. Regarding this, VOC emissions have been reported as emission factors (*i.e*., amount of VOC emitted per weight of waste treated) or from a Life Cycle Assessment (LCA) perspective, since their contribution is mainly related to the Photochemical Oxidation Potential (POP). Emission factor values of 1.70 and 0.59 kg of VOC Mg^−1^ of OFMSW treated were reported by Baky and Eriksson [15] and Smet [3] from studies at laboratory scale, while Cadena *et al*. [16] found 0.2 and 7.3 kg VOC Mg^−1^ of OFMSW treated in two different full scale composting plants using different technologies. In LCA studies, emissions of VOC to the atmosphere are expressed in kg ethylene equivalent/kg of emission and included in the POP category [17].

The presence of VOCs in gaseous emissions from waste treatment plants has been reported as total VOCs emitted [16–19], as concentration of different families of VOCs [14,19] or as individual VOC concentrations [3,20]. Table 1 summarizes some values reported for VOC emissions in biological treatment processes of organic wastes by different authors. Some values in Table 1 are reported as a range of VOC concentrations obtained in a same plant during different sampling days or as different values obtained by the same authors in different plants. As seen in Table 1, values of VOC concentration are very variable, even for the same facility and the same type of waste. In the case of total terpenes, for instance, values reported in Table 1 for an effluent of a biofilter in a composting plant range between 20 and 12,350 μg/m^3^, while the same type of compounds for an anaerobic digestion plant show concentrations between 683 and 4,750 ppbv for non-treated OFMSW and 414 and 1,151 ppbv for the anaerobically digested residue. As a general trend observed from the wastes and treatments reported, the compounds with the highest presence in the emissions are terpenes and alcohols followed by ketones.

Sulphur compounds, with a relatively low concentration, are significant for their contribution to the odor level. In fact, dimethyl sulfide and dimethyl disulfide together with carbon disulfide and methyl and ethyl mercaptan have been found in gaseous emissions from aerobic and anaerobic waste treatments. Actually, these compounds are very common in organic waste decomposition processes, originating mainly in the microbial degradation of sulphur-containing amino acids found in proteins [3,23].

Regarding nitrogen compounds, there is an important difference within values reported resulting from the analysis of samples using GC-MS (Gas Chromatography-Mass Spectrometry) and gas detection tubes, with those obtained with tubes being higher (plants studied in both cases correspond to different treatments). It is clear that both analytical methodologies need a deeper and more complete comparison to be fully reliable being, at present, GC-MS the most powerful tool to quantify VOC emissions.

There are also other compounds that have not been listed in Table 1 but have been detected by some authors in low concentrations. This is the case, for instance, of halogenated and aromatic compounds such as trichlorofluoromethane, 1,1,1-trichloroethane, dicloromethane, 1,3-dichlorobenzene, naphthalene or *p*-isopropyltoluene [2,14,20] or other terpenes such as camphene or thujone [3,20].

The presence of VOCs has also been investigated in landfill gas and ambient air surrounding landfill facilities [24,25]. Aromatic VOCs (benzene, toluene, ethylbenzene and xylene) and reduced sulphur compounds (RSC) were found in these studies, with their concentration and relative percentages in landfill gas being strongly dependant on landfill aging [26]. Among the RSCs found in these emissions, hydrogen sulfide was predominant both in emissions from active and inactive landfill stages [27]. In addition to this, the conditions of the waste in the landfill, which are mainly anaerobic, must be related to the composition of the emitted gas [9].

### VOC and Odors

The presence of odors is the main concern associated with VOC emissions and it has been investigated by a wide number of researchers. 2-butanone, α-pinene, tetrachloroethylene, dimethyl disulfide, β-pinene, limonene, phenol and benzoic acid were included in the study of Bruno *et al*. [28] as representative compounds of some important classes related to high odor impact.

Pierucci *et al*. [12] found that odorimetric tests (olfactometry measurements) were in agreement with GC-MS analysis of VOCs, especially for terpenes when VOCs and odors from MSW composting were monitored. Defoer *et al*. [20] concluded that the relationship between chemical (GC-MS measurements) and odor concentration (olfactometry) is specific for each type of odor and cannot be generalized. These authors established a good linear relationship between odor concentration and total VOC concentration at a biofilter output during the composting of vegetable, fruit and garden wastes. Odor concentration was also well correlated with esters and ketones content. However, in an animal rendering plant, VOCs emitted from the biofilter were a poor indicator of the odor level. In this case, the best relationship was found for compounds containing organic sulphur.

Mao *et al*. [22] determined ammonia, amines, dimethyl sulfide and acetic acid as the responsible for most odors in food waste composting plants compared to numerous VOC. These authors identified 29 compounds in the odor from this type of waste treatment plants although no correlation attempt between chemical analyses and odor concentration measurements was reported.

Tsai *et al*. [21] investigated the relationship of the critical odorants from food waste composting plants with their human olfactory effect (olfactometry results). These authors found six critical odorants: ethylbenzene, dimethyl sulfide, trimethylamine, *p*-cymene, ammonia and acetic acid. Correlations found were different when low or high concentrations of these compounds were considered. For ethylbenzene, dimethyl sulfide, trimethylamine and *p*-cymene, which presented a very low olfactory threshold (0.002 ppm), a linear relationship between concentration and odor was only found for concentration values within the 0.25–100 ppm range. A linear correlation was also found for odors and acetic acid (in the 0.1–50 ppm range), while it was not possible for ammonia when concentrations within 5 and 100 ppm were considered.

On the other hand, D’Imporzano *et al*. [29] and Orzi *et al*. [19] tried to correlate VOC emissions with odors measured by an electronic nose and also with the biological activity during the biological treatment process. D’Imporzano *et al*. [29] found a good correlation between odor molecules detected by the electronic nose and the Dynamic Respiration Index (DRI), used as a measure of the biological activity during a food-waste composting process at pilot scale. Even if an adequate oxygen concentration was maintained during the biological treatment process, anaerobic conditions were developed during the highest microbial activity stage resulting in a high level of sulphur compounds, methane and hydrogen in the outlet gas stream. Orzi *et al*. [19] studied odors (measured by the electronic nose and by olfactometry) and VOC concentration (using GC-MS) also relating them to the biological activity (measured using aerobic and anaerobic indices) in an anaerobic digestion treatment plant processing the organic fraction of municipal solid waste. These authors state that as the biological stability increases (during the entire anaerobic digestion process represented by the sampling points, *i.e*., not digested waste, digested waste and post-digested waste) odor emissions measured by olfactometry decrease, although no correlation between total VOC concentration and olfactometry measurements could be established. Data obtained from the electronic nose measures showed that odor reduction due to the increment of biological stability was accompanied by a change in the organic compounds present in air samples. Further measurements using GC-MS confirmed these results as VOC mainly present in air samples obtained for the air surrounding fresh waste were terpenes (61%), alcohols (18%) and esters (9%), while air samples from digested waste still presented a high presence of terpenes (51%) and carbonyl compounds (40%), being these same compounds predominant in post-digested waste (58% of terpenes and 34% of carbonyl compounds). Regarding this point, it is evident that a reliable correlation between the odor values obtained from electronic noses or olfactometry measurements and the chemical composition determined by CG-MS still has not been established. Consequently, no international consensus about the suitability of these techniques has been reached.

## 2. VOC Sampling and Detection

The main sources of VOCs and other gaseous contaminants in composting plants are area emission sources. Composting or, in general, waste treatment plants, can be completely confined with process emissions treated through scrubbers and/or biofilters or completely open, where the composting process takes place commonly in windrows (static, aerated and/or turned), or a mixture of both situations. Then, biofilter and composting windrows surfaces are the sources of VOCs. In addition, emissions from reception and storage areas should be considered if not confined. Also the emissions of VOCs during some pre (waste conditioning) and post treatment (compost sieving) operations can be significant. In fact, Albretch *et al*. [30] stated that odors emission during turning, sieving and shredding may exceed those from biofilters. Also, Pagans *et al*. [11] showed a continuous emissions of VOCs from the biofilter even when this was not working. In anaerobic digestion treatment plants, area emission sources also exist as a focus of VOCs (biofilters external surfaces), together with some point emission sources, such as safety flares for biogas.

### 2.1. VOC Sampling: Obtaining Representative Samples from Area Sources

As can be deduced from the nature of the emitting surfaces, obtaining representative and comparable samples of gaseous emissions is not straightforward. In addition, no impact on the characteristics and conditions of the source should be caused when sampling and no influence of the equipment used on the sample should be guaranteed [31]. In the case of area sources, it is generally very difficult to cover the entire emission area during sampling. Representative sampling sites have to be established, but there are no regulations on how to select the sites [31]. Hudson *et al*. [32] located odor sampling points according to a regular array at the surface of different shape piggery anaerobic treatment ponds, being the number of points related to the pond surface area. Odor monitoring was conducted at different periods during a year to take into consideration the possible seasonal variations in the waste characteristics and climatic conditions. Average values were calculated for each pond. A wind tunnel device was used to collect air samples forcing carbon filtered ambient air into the tunnel and measuring air velocity with a hot wire anemometer. Sironi *et al*. [33] also used the wind tunnel to obtain air samples for odor analysis at different points of mechanical-biological treatment plants of MSW. These authors also reported the use of a flux chamber and a static hood in the study of a composting plant [34]. The flux chamber was positioned on the heaps during sufficient time to reach equilibrium conditions between the gas and the solid phase. Afterwards, the air sample was collected sucking the air by means of a depression pump inside Nalophan™ bags. The static hood was used to obtain air samples on a biofilter surface with the function of isolating the sampling point from the external conditions and to channel the air stream in a separated stack. To obtain representative air samples from a biofilter surface, Defoer *et al*. [20] covered the entire surface with a polyethylene foil fixing three of the four sides on the biofilter material. Gaseous samples were obtained by a PTFE tube from the open side of the covering foil.

Cadena *et al*. [35] proposed a methodology to obtain gaseous emission samples from windrows and biofilters surfaces in waste treatment plants. The methodology consists of determining the air velocity and the concentration of selected compounds in a matrix of points on the emitting surface defined on the basis of the emitting surface dimensions (although different sampling points at the top and both sides of the composting windrows were considered, maximum emission was always found at the top). Air velocity was directly measured at field by means of a thermo anemometer, which limited sensitivity was overcome using a home-made Venturi, protected by a plastic box [18,36]. Air samples were collected in Tedlar™ bags for VOC analysis. The product of VOC concentration (mg·m^−3^) and air velocity (m·s^−1^) results in VOC mass flow released per windrow surface area unit (mg·s^−1^·m^−2^). Measures of VOC emissions were repeated at different days. Data obtained from emission measurements during a single sampling day were represented in a three dimensional graph with windrow length and perimeter in x and y axes respectively (or alternatively the biofilter dimensions). VOC mass flow values per square meter were placed in the z-axis to obtain an emission surface. The three dimension emission surface was then projected in a two-dimension graph (windrow perimeter at x-axis and windrow length at y-axis). Multiplying the pollutant mass flow per area unit by the corresponding area in the graph resulted in the compound mass flow and the sum of the different quantities obtained corresponds to the total mass flow of VOC (g·s^−1^). VOCs were determined as total carbon concentration by GC using a FID (flame ionization detector).

### 2.2. VOC Samples Preparation and Detection

Detection and quantification of VOCs has been performed by different techniques. Although the gas chromatography is preferred by a number of researchers, gas detection tubes, electronic noses and olfactometry have also been used for VOC determination in gaseous samples.

#### VOC Detection by Gas Chromatography

The most common technique used in VOC detection and quantification is gas chromatography. However, in most of the cases due to the low concentration of these compounds in the samples to analyze, a pre-concentration step is needed to ensure the complete detection of all VOCs present and the accuracy of the analysis. Different materials and settings are used with this purpose. Defoer *et al*. [20] pre-concentrated gaseous samples from a biofilter surface by sorption onto Tenax TA prior to their analysis by GC-MS preceded by thermal desorption (220 °C) and cold trapping using liquid nitrogen. A 60 m dimethylpolysiloxane column (film thickness 1.5 μm, internal diameter 0.53 mm) was used. The GC was provided with a splitter that allows analyzing part of the eluate in a FID detector for VOC quantification and the other part in a MS for VOC identification. Tolvanen *et al*. [13] collected air samples from a composting hall in a full scale plant into Tenax-GR adsorption tubes using an air pump. VOC analyses were performed in the laboratory with a thermal desorption/gas chromatograph/mass spectrometer system in combination with simultaneous sniffing. Desorption temperature was maintained during 10 min at 250 °C and during this time desorbed compounds were concentrated in a cold trap (−120 °C). The GC was equipped with a double column system (HP 5, 30 m, 1 μm film) with compounds eluted from one column diverted to MS and those from the other column sniffed. Concentration of single VOCs (25 different compounds) were calculated according to an external standard. Adsorption onto Tenax cartridges was also suggested and used by Bruno *et al*. [28], who state that the low water affinity of this material and the high number of substances that is able to adsorb make them an ideal material for the sampling of heterogeneous organic compounds such as those produced in waste treatment plants. These authors use diffusive samplers made up of a cylindrical adsorbing cartridge coaxially put inside a cylindrical diffusive body (polycarbonate and microporous polyethylene) holding a known amount of Tenax. The analysis of the compounds was undertaken by GC-MS with previous desorption.

Komilis *et al*. [14] used activated coconut charcoal traps to obtain gaseous samples for VOC determination in composting experiments at laboratory level. VOCs were then extracted with high purity carbon disulfide under agitation and then analyzed using GC-FID (60 m × 0.32 mm SPB-5 capillary column). GC-MS determinations were performed using a 30 m × 0.25 mm HP-5MS capillary column. Pierucci *et al*. [12] collected VOCs from the headspace of MSW samples (kept in vials and heated to 85 °C) and also from air bag samples using solid phase microextraction (SPME). Different fibers were compared presenting different coating materials and a three-phase fiber polydimethylsiloxan-carboxen-divinylbenzene (PDMS-Carboxen-DVB) was finally selected. GC-MS was then used for analysis and VOC identification. This type of fibers was also used by Orzi *et al*. [19] when determining odors and VOCs in an anaerobic digestion treatment plant. In this case, solid samples were carried to the laboratory and gaseous samples collected using a flux chamber. The waste sample was put in a tray container and covered with a Plexiglas chamber continuously flushed with a known air flow. Gas samples were collected from the output port of the chamber in Nalophane bags. The SPME fiber was exposed in the bags for VOC adsorption. VOCs were desorbed for GC-MS analysis exposing the fiber in the GC injection port for 3 min at 250 °C. Some authors have also used gas chromatography without samples pre-concentration to determine VOC emissions from the composting process of several wastes, although they are mainly focused on the composting of olive oil wastes and their effect on carbon sequestration in soil [37,38].

#### Other VOC Detection Techniques

In addition to the use of GC-MS for VOC determination, there is the possibility of using gas detection tubes. In fact, Mao *et al*. [22] and Tsai *et al*. [21] present their results on VOC emissions from food waste composting plants using both analytical procedures. Mao *et al*. [22] also used two different brands for gas detection tubes resulting in differences in analytical results around 20%. Gas detection tubes permit the measure of many critical odorants when these are present in relatively high concentrations in a quicker and cheaper way when compared to GC-MS. For this reason, gas detection tubes can be a practical tool for the self management of odor problems in waste treatment plants. Gas samples were obtained in these studies at different points of the composting plants at 1.2 m of the floor using a pump to collect samples for GC-MS analysis in Tedlar bags and the specific gas sampling pump provided with the gas detection tubes.

The electronic nose (EN) has been also used for odor determination and VOC families’ identification [19,29]. An electronic nose is configured by a number of sensors each of them sensitive to a grouped class of compounds. In the case of the authors cited, the electronic nose used was equipped with ten sensors corresponding to S1: aromatic compounds, S2: polar compounds and nitrogen oxides, S3: aromatic compounds, ketones and aldehydes, S4: H_2_, S5: low polarity aromatic and alkane compounds, S6: methane, S7: sulphur compounds and terpenes, S8: alcohols, ketones and partially aromatic compounds, S9: sulphur containing and aromatic compounds and S10: methane at high concentration (PEN2 electronic nose, Airsense Analytics, Schwerin, Germany). Although very attractive, this methodology still needs a scientific validation for a large number of odors and sources. As stated above in this paper (Section 1.1), olfactometric techniques have also been used for VOC measurement although their suitability for VOC quantification still has not been demonstrated.

## 3. Treatment Approaches for Gaseous VOC Emissions

Although gaseous emissions can be directly released to the atmosphere in non-confined waste biological treatment facilities [16], effective gas treatment equipment is usually required in most cases, especially when the distance to inhabited areas is low [18]. As can be deduced from the numerous VOC detected in gaseous emissions from waste treatment plants reported above, the selection of the adequate equipment for gaseous pollutants abatement in these installations is not an easy task. There is no specific technology developed for this application and normally a combination of the available options must be studied. Adsorption, absorption (scrubbing), thermal/catalytic oxidation and biological treatments have been used for VOC removal in gaseous emissions [39]. Adsorption and scrubbing involve the generation of a liquid or solid effluent containing the contaminants removed from gaseous flows and thus further treatment will be needed for these waste flows. Sulphur oxides could be generated in a thermal treatment depending on VOCs present in the gaseous effluent from a waste treatment plant. Although presenting acceptable VOC removal rates, physico-chemical treatments involve higher economical costs than biological treatments.

Biological treatments of waste gases are based on the use of naturally selected microbial strains capable of contaminant removal which act as carbon source, energy source or both [39]. Different types of bioreactors are used in VOC contaminated gas treatment including biofilters, biotrickling filters and bioscrubbers. Contaminant removal mechanisms are similar in these bioreactors differences relying in the use of microorganisms, either suspended or immobilized, packing media, pollutant concentration and others [40]. Among the available options, biofiltration is the most commonly used technology to reduce emissions from the biological waste treatment processes [41]. At large facilities biofiltration is usually preceded by an absorption step under acidic conditions, mainly to remove ammonia that is produced in large amounts during the composting process and that can inhibit the biological activity of the biofilter [42]. Biofiltration is considered economically and environmentally viable and a suitable technology in terms of waste recycling and filtering effect [43].

**Figure 1.**
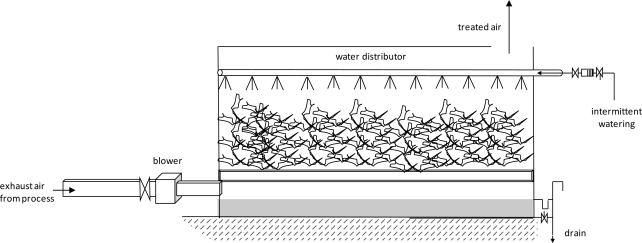
Scheme of a biofilter.

Biofilters are a type of bioreactors in which a humidified polluted air stream is passed through a porous packed bed on which a mixed culture of pollutant-degrading organisms is immobilized. Generally the pollutants in the air flow are transported from the gaseous phase to the microbial biofilm (through liquid phase or moisture) where the biological oxidation of VOCs occurs [40]. The by-products of biological oxidation are water, carbon dioxide, mineral salts, some volatile organic compounds and microbial biomass [44]. Organic packing materials are commonly used such as wood chips mixed with compost or other bulking materials used in the composting process. Figure 1 shows a scheme of a biofilter. The performance of a biofilter is not uniform and it is influenced by several important variables such as the composition of the gas, the packing material, nutrient supply, temperature, pH, pressure drop and gas residence time [45]. Maximum elimination capacities of 100–120 g·m^−3^·h^−1^ have been reported for typical biofilters colonized with bacteria [46]. These authors developed a method to assess biofilters performance for a wide range of VOCs with different air/water partition coefficients. Some authors indicate that the addition of activated carbon improves the biofilter degradation capacity [40]. However, the prediction of the biofilters efficiency in the removal of a mixture of VOCs such as the one present in the exhaust gases of a waste treatment plant is difficult, being limited by the solubility and biodegradability of pollutants.

Pagans *et al*. [11] reported biofilter removal efficiencies up to 97% for VOCs generated in the composting process of different organic wastes at pilot plant scale with loading rates ranging from 0.55 to 40 g·C·m^−3^·biofilter·h^−1^. Removal efficiencies were highly dependent on the composted waste. The same authors also pointed a biofilter basal emission of VOCs of approximately 50 mg of C·m^−3^. However, in the literature, VOC biofiltration is frequently studied in laboratory scale biofilters using synthetic gases with two or three mixed compounds or even a single compound [47,48], which can be very different from those conditions found in full-scale facilities, as previously commented.

Another aspect to comment in biofilter operation and maintenance at an industrial level is the need for attention to packing material needs for a correct performance of the gas treatment equipment. In fact, watering of the material is needed, as well as a periodic replacement of the biofilter material. Colón *et al*. [18] reported real data on two full scale biofilters treating gases from the OFMSW composting process during filtering material replacement. Old biofilter material showed lower removal efficiencies than the new material. The average VOC removal efficiencies with the old material for the two biofilters studied were 42 and 65%, whereas the average values of all data with new material were 74 and 71%. These efficiencies correspond to average elimination capacities of 11 and 8.6 g·C·m^−3^ biofilter h^−1^ for the old material and 17.1 and 27 g·C·m^−3^·biofilter·h^−1^ for the new material. These results indicate that the biofilter performance was improved as a result of material replacement. In one of the two biofilters the authors observed the pattern reported by Devinny *et al*. [45] in relation to VOC removal in biofilters: a first stage of dominance of the adsorption process followed by a decrease of the removal efficiency attributable to the saturation of the adsorption/absorption capacity and to the microorganisms acclimation period and, finally, an increase in the removal efficiency because of the biodegradation dominance [49]. For the second biofilter, the removal efficiency followed a similar pattern to the applied VOC loading rate during the entire period of the study (before and after biofilter material replacement). Even though biofilters are widely implemented in full scale treatment plants for organic waste treatment facilities, the complete removal of VOC is difficult to achieve [20]. Proper maintenance operations are thus needed to maximize biofilter performance.

## 4. Conclusions

VOCs present in gaseous emissions of biological treatment facilities of organic wastes have been investigated and characterized by different researchers. The main VOCs related to organic waste handling and treatment are terpenes, although ketones, alcohols and organic acids have also an important contribution to the total VOC content measured. The presence of sulphur and nitrogen compounds has to be also highlighted. Different relationships have been established between VOC concentration and odor level on specific situations and for a specific range of VOCs and odor concentrations. There is no universal correlation covering the whole concentration range and the entire group of VOC families.

The analysis of VOCs is mainly performed by GC-MS although other methods such as gas detection tubes or electronic noses have been used in some cases. GC-MS appears as the most reliable method covering a wide range of VOC concentrations and being capable to identify and quantify a large number of compounds from different families. Gas detection tubes permit the measurement of many compounds present at relatively high concentrations. As an economical and quick method, it can be useful for the self management of gaseous emissions in waste treatment plants. Electronic noses permit the characterization of the families of VOC present in a gaseous sample and the comparison between VOC emissions from different points of a treatment plant of different installations, although they must be considered a first approach.

Different methods for air emissions sampling in waste treatment plants have been proposed. The lack of homogeneity among these methods is one of the drawbacks in VOC emission measurement from this type of facilities.

Finally, it must be highlighted that biofiltration is the gaseous pollutant abatement technology that is most widely used in organic waste treatment plants for VOC removal.

## Figures and Tables

**Table 1.** VOC emissions from waste treatment plants.

**Compound**	**Concentration**	**Units**	**Waste**	**Type of plant/measurement area**	**Reference**
*Hydrocarbons (total)*	30/560	μg m^−3^	VFG^1^	Effluent of biofilter	[20]
Pentene	nd/26.20 nd/75	ppb μg m^−3^	Food waste Food waste	2 composting plants-Composting hall 2 composting plants-Composting hall	[21] [22]
Hexene	8.02/16.13 28/55	ppb ppb	Food waste Food waste	2 composting plants-Composting hall 2 composting plants-Composting hall	[21] [22]
Benzene	1.04/17.52 3/56	ppb ppb	Food waste Food waste	2 composting plants-Composting hall 2 composting plants-Composting hall	[21] [22]
Toluene	5.24/16.90 20/64	ppb ppb	Food waste Food waste	2 composting plants-Composting hall 2 composting plants-Composting hall	[21] [22]
Ethylbenzene	1.29/6.74 6/29	ppb ppb	Food waste Food waste	2 composting plants-Composting hall 2 composting plants-Composting hall	[21] [22]
*p,m*-Xylene	1.95/10.56 8/46	ppb ppb	Food waste Food waste	2 composting plants-Composting hall 2 composting plants-Composting hall	[21] [22]
Styrene	68.50/113.58 291/482	ppb ppb	Food waste Food waste	2 composting plants-Composting hall 2 composting plants-Composting hall	[21] [22]
*o*-Xylene	1.00/8.01 360/842	ppb ppb	Food waste Food waste	2 composting plants-Composting hall 2 composting plants-Composting hall	[21] [22]
Aliphatic Hydrocarbons (total)	2.07–30.2/0.48–14	ppbv	OFMSW	Anaerobic digestion plant F/D^2^	[19]
Aromatic Hydrocarbons (total)	19.3–39.1/22.3–55.1	ppbv	OFMSW	Anaerobic digestion plant F/D^2^	[19]
*Ketones (total)*	nd-2820	μg m^−3^	VFG^1^	Effluent of biofilter	[20]
Acetone	211.17/187.15 500/443	ppb ppb	Food waste Food waste	2 composting plants-Composting hall 2 composting plants-Composting hall	[21] [22]
Butanone	15.17/10.34 45/30 0.002–0.56 12–337/311–556	ppb ppb mg·m^−3^ ppbv	Food waste Food waste Food waste OFMSW	2 composting plants-Composting hall 2 composting plants-Composting hall Composting plant-Composting hall Anaerobic digestion plant F/D^2^	[21] [22] [13] [19]
2,3-Butanedione	0.009–1.15	mg·m^−3^	Food waste	Composting plant-Composting hall	[13]
*Alcohols (total)*	nd-4150 413–762/38.5–82.6	μg·m^−3^ ppbv	VFG^1^ OFMSW	Effluent of biofilter Anaerobic digestion plant F/D^2^	[20] [19]
2-Butanol	nd-0.69 105–208/17–75	mg·m^−3^ ppbv	Food waste OFMSW	Composting plant-Composting hall Anaerobic digestion plant F/D^2^	[13] [19]
3-Methyl-1-butanol	0.02–0.13	mg·m^−3^	Food waste	Composting plant-Composting hall	[13]
Ethanol	176–365/4–7	ppbv	OFMSW	Anaerobic digestion plant F/D^2^	[19]
Propanol	36–121/0	ppbv	OFMSW	Anaerobic digestion plant F/D^2^	[19]
*Aldehydes (total)*	nd-3460	μg·m^−3^	VFG^1^	Effluent of biofilter	[20]
3-Methylbutanal	0.003–0.03	mg·m^−3^	Food waste	Composting plant-Composting hall	[13]
*Ethers (total)*	nd-270 0–78.8/1.72–6.4	μg·m^−3^ ppbv	VFG^1^ OFMSW	Effluent of biofilter Anaerobic digestion plant F/D^2^	[20] [19]
*Esters (total)*	nd-3270	μg·m^−3^	VFG^1^	Effluent of biofilter	[20]
	224.3–355.3/0.00–12.7	ppbv	OFMSW	Anaerobic digestion plant F/D^2^	[19]
Methyl acetate	1.31/5.24 4/16 0.003–0.11 55–158	ppb ppb mg·m^−3^ ppbv	Food waste Food waste Food waste OFMSW	2 composting plants-Composting hall 2 composting plants-Composting hall Composting plant-Composting hall Anaerobic digestion plant F/D^2^	[21] [22] [13] [19]
Ethyl acetate	2.49/nd 9/nd 0.02–0.49	ppb ppb mg·m^−3^	Food waste Food waste Food waste	2 composting plants-Composting hall 2 composting plants-Composting hall Composting plant-Composting hall	[21] [22] [13]
Ethyl hexanate	0.001–0.07	mg·m^−3^	Food waste	Composting plant-Composting hall	[13]
*Terpenes (total)*	20–12350 683–4750/414–1151	μg·m^−3^ ppbv	VFG^1^ OFMSW	Effluent of biofilter Anaerobic digestion plant F/D^2^	[20] [19]
α-Pinene	nd/2.56 nd/14 0.002–0.12 26–80/0	ppb ppb mg·m^−3^ ppbv	Food waste Food waste Food waste OFMSW	2 composting plants-Composting hall 2 composting plants-Composting hall Composting plant-Composting hall Anaerobic digestion plant F/D^2^	[21] [22] [13] [19]
β-Pinene	7.29/7.71 41/43 0.02–0.04	ppb ppb mg·m^−3^	Food waste Food waste Food waste	2 composting plants-Composting hall 2 composting plants-Composting hall Composting plant-Composting hall	[21] [22] [13]
Limonene	43.29/66.29 240/368 0.02–1.40 433–4389/178–920	ppb ppb mg·m^−3^ ppbv	Food waste Food waste Food waste OFMSW	2 composting plants-Composting hall 2 composting plants-Composting hall Composting plant-Composting hall Anaerobic digestion plant F/D^2^	[21] [22] [13] [19]
*p*-Cymene	9.00/11.57 49/63 64–80/53–134	ppb ppb ppbv	Food waste Food waste OFMSW	2 composting plants-Composting hall 2 composting plants-Composting hall Anaerobic digestion plant F/D^2^	[21] [22] [19]
β-Myrcene	0.04–0.11	mg·m^−3^	Food waste	Composting plant-Composting hall	[13]
3-Carene	0.007–0.03	mg·m^−3^	Food waste	Composting plant-Composting hall	[13]
*Nitrogen compounds (total)*	1000/30000^a^ 0.00–3.42/0.00–7.11	ppb ppbv	Food waste OFMSW	2 composting plants-Composting hall Anaerobic digestion plant F/D^2^	[21] [19]
*Sulphur compounds (total)*	nd-290 0–79.6/0–8.1	μg·m^−3^ ppbv	VFG^1^ OFMSW	Effluent of biofilter Anaerobic digestion plant F/D^2^	[20] [19]
Dimethyl sulfide	nd/300^a^ nd-0.003	ppb mg·m^−3^	Food waste Food waste	2 composting plants-Composting hall Composting plant-Composting hall	[21] [13]
Dimethyl disulfide	0.001–0.004	mg·m^−3^	Food waste	Composting plant-Composting hall	[13]
*Halogenated compounds (total)*	nd-40 0–0.08/0	μg·m^−3^ ppbv	VFG^1^ OFMSW	Effluent of biofilter Anaerobic digestion plant F/D^2^	[20] [19]
*Carboxylic acids*	0–164.7/0	ppbv	OFMSW	Anaerobic digestion plant F/D^2^	[19]
Acetic acid	250/nd^a^ 0.05–1.02	ppb mg·m^−3^	Food waste Food waste	2 composting plants-Composting hall Composting plant-Composting hall	[21] [13]
Butanoic acid	0.0005–0.14	mg·m^−3^	Food waste	Composting plant-Composting hall	[13]
Furans	nd-270	μg·m^−3^	VFG^1^	Effluent of biofilter	[20]
TOTAL VOC	0.71–10.10 1672–6005/1178–1694 612–63 0.08–1.4	mg·m^−3^ ppbv mg·C·m^−3^ g·C·m^−3^	Food waste OFMSW OFMSW OFMSW	Composting plant-Composting hall Anaerobic digestion plant F/D^2^ Composting pilot scale reactor Composting plant (in vessel)-biofilter input	[13] [19] [11] [18]

1 VFG: vegetable, fruit and garden waste.

2 F/D: fresh, non digested waste/digested waste.

a Measured with gas detector tubes. nd: not detected.

The symbol “-“ in concentration values means the range of VOC detected. The symbol “/“ in concentration values means the results of different plants or measurement areas in the same plant (see fifth column for specific locations).
